# Applications of Nature-Inspired Water Cycle Algorithm in Antenna Design and Array Synthesis

**DOI:** 10.3390/s26092724

**Published:** 2026-04-28

**Authors:** Yixi Wei, Yanhong Xu, Weiwei Wang, Anyi Wang, Jingwei Xu, Kwai-Man Luk

**Affiliations:** 1 School of Communication and Information Engineering, Xi’an University of Science and Technology, Xi’an 710054, China; 25207040058@stu.xust.edu.cn (Y.W.); wanganyi@xust.edu.cn (A.W.); 2 China Academy of Space Technology, Xi’an 710100, China; wangweiwei@cast504.com; 3 School of Electronic Engineering, Xidian University, Xi’an 710071, China; jwxu@xidian.edu.cn; 4 State Key Laboratory of Terahertz and Millimeter Waves, City University of Hong Kong, Hong Kong SAR, China; eekmluk@cityu.edu.hk

**Keywords:** nature-inspired algorithm, electromagnetic (EM) problem, wideband antenna design within a given space, array pattern synthesis in scenario of strong mutual coupling

## Abstract

Continuous introduction of advanced optimization algorithms promotes the development of electromagnetic (EM) technology in radar and communication systems. Wideband antenna design within a given space and wideband array pattern synthesis, especially in the scenario of strong mutual coupling, are two typical challenging electromagnetic problems. In this paper, a nature-inspired algorithm, i.e., the water cycle algorithm (WCA), is introduced to resolve the above two EM problems. Two typical wideband antennas, i.e., the dual-band E-shaped microstrip antenna and the typical magnetoelectric (ME) dipole antenna, are designed on the basis of the established WCA-based antenna design scheme. Compared with the well-known algorithms that have been introduced in antenna design, including the differential evolution (DE) algorithm and the gray wolf optimizer (GWO), better results can be achieved with WCA. In the sequel, a WCA-based low peak sidelobe level (PSLL) pattern synthesis is implemented based on a uniformly spaced 27-element folded fractal ME dipole array antenna with mutual coupling as high as −10 dB, the results of which further validate the superiority of WCA in array pattern synthesis and demonstrate the value of this application innovation.

## 1. Introduction

Antenna design and array antenna synthesis are two typical electromagnetic (EM) problems for radar and communication systems [1,2], each of which is usually a nonlinear and multi-dimensional problem difficult to solve using conventional gradient-based approaches [3,4]. Broadband connectivity in 6G [5] enables numerous technologies, including integrated sensing and communication (ISC) [6], and integrated radar and communication (IRC) [7], where wideband antenna design within a given space and array pattern synthesis in case of strong mutual coupling (often neglected by many reported works, e.g., [8,9]) are still challenging to improve the performance of electric systems.

To maximize the antenna performance within the same space or enhance the performance of an array antenna, numerous algorithms have been explored to solve such EM problems [10,11,12,13]. Among these algorithms, nature-inspired optimization algorithms play an important role. The genetic algorithm (GA) is one of the earliest nature-inspired algorithms introduced to and still widely utilized in antenna design [14,15,16] and array antenna synthesis [17,18,19]. Specifically, designs of wire antennas are presented in [14,15], and the design of miniaturized and reconfigurable Internet of Things (IoT) antennas is revealed in [16]. The differential evolution (DE) algorithm is another representative nature-inspired algorithm that has been utilized in EM problems [20,21,22,23]. Specifically, the performance comparisons of various DE algorithms in designing a microstrip antenna with a probe/cross-aperture-coupled feed are provided in [23]. Particle swarm optimization (PSO) is another typical nature-inspired algorithm that has been widely applied in the optimization of EM problems [24,25,26,27], where typical investigations of the PSO in antenna design and beampattern synthesis are provided. Apart from the above three typical algorithms, other nature-inspired algorithms including wind driven optimization (WDO) [28], spider monkey optimization (SMO) [29], fruit-fly optimization (FFO) [30], bat algorithm (BA) [31], and adaptive immune annealing algorithm (AIAA) [32] have been utilized to successfully handle a wide range of EM problems in recent years. From the above descriptions, it can be concluded that the nature-inspired optimization algorithm is a powerful tool to solve complicated antenna design and array synthesis problems. The continuous introduction of nature-inspired optimization algorithms is a non-negligible factor that promotes the development of EM technology.

Based on the phenomenon of the water cycle in nature, where rivers and streams flow downhill towards the sea, the water cycle algorithm (WCA) is proposed by Eskandar [33], which has the advantages of a fast optimization rate and high iterative accuracy, and is a good candidate to solve constrained engineering optimization problems [34]. At present, only limited attempts have been conducted to investigate the effectiveness of the applications of WCA in antenna and array designs [35,36,37]. In this paper, we focus on an application innovation by investigating the performance of WCA in the design of wideband antennas and array pattern synthesis. The following provides a brief summarization of the main contributions of this application innovation.

(1) The WCA-based antenna optimization scheme is established, and its effectiveness is first verified by designing two representative wideband antennas. Better results are achieved compared with well-known metaheuristic algorithms, such as GA, PSO, DE, and GWO.
(2) Based on the above optimization scheme, a bandwidth-enhanced magnetoelectric (ME) dipole antenna is designed with a simple structure. A bandwidth of 92.4% ranging from 1.41 GHz to 3.83 GHz is achieved, which further demonstrates the effectiveness of WCA in antenna design.
(3) Then the WCA is applied in low peak sidelobe level (low-PSLL) pattern synthesis based on a uniformly spaced 27-element folded fractal ME dipole array antenna. By incorporating the mutual coupling effects into the fitness function to construct a more accurate electromagnetic forward model, numerical results demonstrate that the WCA exhibits excellent optimization performance in such practical strong coupling scenarios.

The remainder of this paper is structured as follows. Section 2 briefly introduces the WCA and establishes the WCA-based antenna optimization scheme. Section 3 provides two representative wideband antenna designs and a bandwidth-enhanced ME dipole antenna design based on WCA. The WCA-based low-PSLL pattern synthesis in the presence of mutual coupling is provided in Section 4, and the conclusion is drawn in Section 5.

## 2. Brief Introduction of WCA and WCA-Based Antenna Optimization Scheme

In this section, the WCA is first introduced, and four typical test functions are adopted to test its performance along with two other typical metaheuristic algorithms, i.e., GA and PSO. Comparisons are provided along with corresponding detailed analysis. Then, the WCA-based antenna optimization scheme is provided.

### 2.1. Brief Introduction of WCA

#### 2.1.1. Initialization and Classification

Initially, WCA generates a population of *K* raindrops, where each is an *M*-dimensional vector in the solution space, i.e.,

(1) $$\mathrm{Total}\;\mathrm{Population}={\left[R_{1},\cdots ,R_{k},\ldots ,R_{K}\right]}^{T}$$

where $R_{k}={\left[R_{k1},\cdots ,R_{km},\cdots ,R_{kM}\right]}^{T}\in R^{M\times 1}$ with the superscript T being the transpose operator, k=1, ⋯, K, and $R_{km}$ is generated by the following equation

(2) $$R_{km}=F_{\min}+rand\times \left(F_{\max}-F_{\min}\right),m=1,\cdots ,M$$

where $F_{\min}$ and $F_{\max}$ are the lower and upper bounds of the optimization problem, respectively. The fitness value of each raindrop is calculated and stored in vector **f** in the order from the smallest to the largest, i.e.,

(3) $$f\;=\left[f_{1},\cdots ,f_{i},\cdots f_{K}\right]$$

where $f_{i}=f\left(R_{i}\right),\;i=1,\cdots ,K$ and $f\left(R_{i}\right)$ denotes the calculation of the fitness value of $R_{i}$. The raindrop, whose fitness value is $f_{1}$, is selected as the sea, i.e., the best solution at the current stage. In the sequel, the $K_{r}$ second-best raindrops are set as rivers. The rest of the raindrops are set as streams, whose population is $K_{s}$ and $K_{s}=K-K_{r}-1$.

#### 2.1.2. Flow Intensity and Population Update

In WCA, the sea and each river absorb the streams depending on the flow intensity. The number of streams $N_{i}$ flowing to the specific river or the sea is determined by Equations (4) and (5). In specific,

(4) $$C_{i}=f_{i}-f_{K_{r}+2}$$

and

(5) $$N_{i}=round\left\{\left|\frac{C_{i}}{\sum_{i=1}^{K_{r}+1}C_{i}}\right|\times K_{s}\right\}\;$$

where $round\left\{\cdot \right\}$ denotes the rounding operator and $\;i=1,\;\cdots ,\;K_{r}+1$. According to (3) and (4), it is obviously seen that $C_{i}<0$. From (4) and (5), it can be concluded that the larger the $C_{i}$ is, the further the corresponding vector is from the optimal solution, and the fewer rivers or streams the corresponding vector needs to connect.

After the population division, the WCA enters into the iterative optimization procedure. A stream flows to the river along the connecting line between them using a randomly chosen distance defined as x, i.e.,

(6) $$x\in \left(0,\;\beta d_{s-r}\right)$$

where x is a distributed random number between 0 and $\beta d_{s-r}$ with $d_{s-r}$ denoting the distance between the current stream and the river, and $\beta \in \left(1,2\right]$. Note that $\beta \in \left(1,2\right]$ enables streams to flow in different directions towards the rivers. Generally, set as β=2. This concept can also be utilized in flowing rivers to the sea. Hence, the new positions for streams and rivers are represented as:

(7a) $$R_{\mathrm{stream}}(t+1)=R_{\mathrm{stream}}(t)+rand\times \beta \times \left(R_{\mathrm{river}}(t)-R_{\mathrm{stream}}(t)\right)$$

(7b) $$R_{\mathrm{stream}}(t+1)=R_{\mathrm{stream}}(t)+rand\times \beta \times \left(R_{sea}(t)-R_{\mathrm{stream}}(t)\right)$$

and

(7c) $$R_{\mathrm{river}}(t+1)=R_{\mathrm{river}}(t)+rand\times \beta \times \left(R_{\mathrm{sea}}(t)-R_{\mathrm{river}}(t)\right)$$

**Figure 1:**
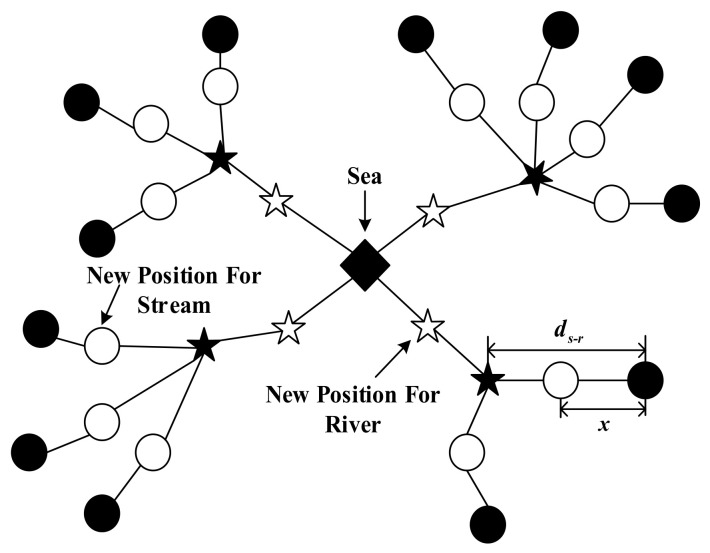
Brief schematic view of WCA [33], where circles, stars, and the diamond correspond to the streams, rivers, and sea, respectively.

Equations (7a) and (7b) separately provide the position renewal process of streams flowing into rivers and the sea, while Equation (7c) depicts the corresponding process of rivers flowing into the sea. If the fitness value of the updated stream (river) is smaller than that of the river (sea), exchange their positions. Figure 1 presents a brief schematic view of WCA.

#### 2.1.3. Evaporation and Rainfall

To enhance the search ability of WCA and prevent it from converging prematurely into local solutions, evaporation and rainfall operations are performed. The evaporation conditions for rivers and streams are

(8a) $$\left\|R_{\mathrm{sea}}-R_{river}^{i}\right\|<\tau_{1},i=1,\cdots ,K_{r}$$

and

(8b) $$\left\|R_{\mathrm{sea}}-R_{\mathrm{stream}}^{j}\right\|<\tau_{2},j=1,\cdots ,K_{s-s}$$

respectively, where $\tau_{1}$ and $\tau_{2}$ are quite small (generally close to 0), and $K_{s-s}$ denotes the number of streams flowing into the sea. $\tau_{1}$ and $\tau_{2}$ control the search intensity near the sea, which is adaptively decreased as

(9a) $$\tau_{1}^{i+1}=\tau_{1}^{i}-\frac{\tau_{1}^{i}}{N_{iter}},i=1,\cdots ,K_{r}$$

and

(9b) $$\tau_{2}^{j+1}=\tau_{2}^{j}-\frac{\tau_{2}^{j}}{N_{iter}},j=1,\cdots ,K_{s-s}$$

where $N_{iter}$ denotes the iteration number.

**Figure 2:**
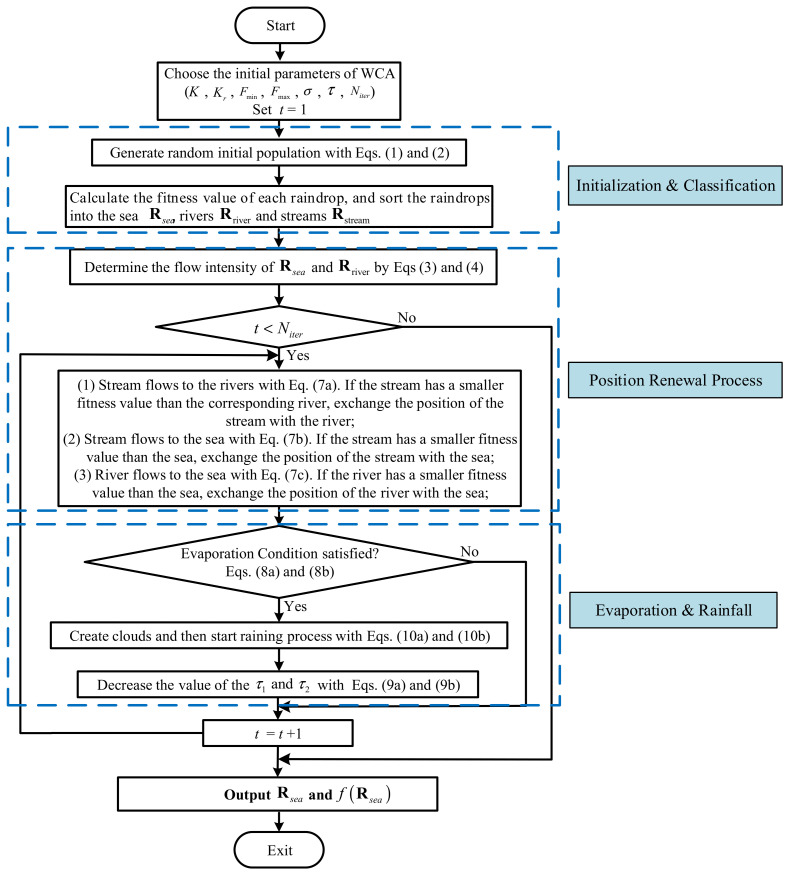
Flowchart of WCA.

When Equation (8a) is satisfied, the streams will be regenerated in the problem space, i.e.,

(10a) $$R_{\mathrm{Stream}m}^{\mathrm{New}}\left(t+1\right)=F_{\min}+rand\times \left(F_{\max}-F_{\min}\right),m=1,\cdots ,M$$

where $R_{\mathrm{Stream}m}^{\mathrm{New}}\left(t+1\right)$ is the *m*th element of $R_{\mathrm{Stream}}^{\mathrm{New}}\left(t+1\right)$. When Equation (8b) is satisfied, the streams will be regenerated near the sea, i.e.,

(10b) $$R_{\mathrm{Stream}}^{\mathrm{New}}\left(t+1\right)=R_{\mathrm{Sea}}\left(t\right)+\sqrt{\sigma }\times v$$

where σ denotes the coefficient showing the range of the searching region near the sea, and $v\in {\mathbb{R}}^{M\times 1}$ is a normally distributed random vector. Actually, σ can be treated as the variance in a mathematical view. Namely, the generated streams are around the sea with variance σ. Generally, a small value of 0.1 can lead the algorithm to search in a small region near the sea. To clearly show the optimization procedure, Figure 2 presents the flowchart of WCA.

#### 2.1.4. Parameter Selection

The optimization performance of the water cycle algorithm is closely related to its parameter settings. To ensure the algorithm achieves stable convergence and strong global exploration ability in optimization problems, this section explains the selection criteria and influence rules of key parameters. β regulates the update step size of streams and rivers, and is generally set to 2 or a value close to 2, so that the updated positions focus on the vicinity of rivers. Moreover, β>1 allows streams to approach rivers from multiple directions, which expands the effective search space and improves the possibility of obtaining better solutions. τ is a tiny value close to 0, which controls the search intensity near the optimal solution and decreases adaptively with the number of iterations, so as to balance the trigger timing of the evaporation operation and the refinement of the local search. σ is used to control the search range of new raindrops near the optimal solution during the rainfall process. An overlarge σ reduces the local optimization accuracy, while an undersized σ leads to an excessively concentrated search range near the optimal solution, which tends to cause premature convergence of the algorithm. Therefore, reasonable parameter selection is essential to achieve better overall optimization performance.

### 2.2. Test on Benchmark Function

The performance of WCA is firstly tested with four typical benchmark functions, i.e., Sphere (F_1_), Schwefel’s Problem 2.22 (F_2_), Ackley’s (F_3_), and Generalized Penalized (F_4_) functions, each of which is selected from the widely used CEC2005 standard test suite and commonly utilized to evaluate the performance of optimization algorithms. Specifically, the Sphere function (F1) is a classic unimodal function with a smooth and convex surface, which is widely adopted to test the convergence speed and optimization precision of the algorithm in a simple search space. Schwefel’s Problem 2.22 (F2) is another typical unimodal function, but it contains nonlinear components. Ackley’s function (F3) is a representative multimodal function with a large number of local optima, which is used to comprehensively evaluate the global exploration capability and the ability to avoid premature convergence. The Generalized Penalized function (F4) is a complex constrained multimodal function with sharp fluctuations and numerous local minima, which can effectively test the stability, robustness, and global optimal searching ability of the algorithm under complex and rugged search environments.

**Figure 3:**
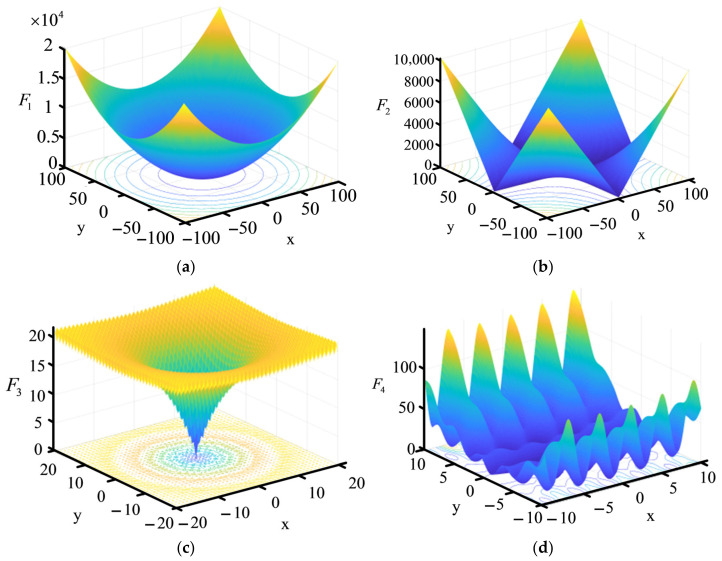
Illustration of the 2-D versions of (**a**) F_1_, (**b**) F_2_, (**c**) F_3_ and (**d**) F_4_.

For comparison, the performances of GA and PSO examined with these four benchmark functions are also provided. The detailed descriptions of these four functions are listed in Table 1, along with the 2-D graphics presented in Figure 3. The F_1_ and F_2_ are unimodal functions, which are used to test the convergence rate and accuracy. The other two are multi-peak functions with multiple minimum values, which are employed to evaluate the ability to search for the global optimal solution. For the sake of comparative fairness, the population size and the maximum iteration number are set to the same value for these three algorithms, i.e., the population size is 100 and the maximum iteration number is 1000. Other parameter settings of each algorithm are as follows: (1) PSO: the social weights are set as $c_{1}=c_{2}=1.5$, and the inertial weight w=0.8; (2) GA: the crossover probability and mutation probability are set to 0.8 and 0.01, respectively; (3) WCA: the number of rivers $K_{r}=3$, $\tau_{1}=\tau_{2}=1\times 10^{-16}$ and β=2. To get statistical results, these three algorithms are independently executed 40 times. The results of the three algorithms, including minimum fitness value (best), maximum fitness value (worst), average fitness value (mean) and standard deviation (std), are provided in Table 2. From Table 2, it can be clearly seen that the WCA not only exhibits higher accuracy and stronger stability compared with PSO and GA, but also significantly accelerates the optimization process with a far superior computation speed. Specifically, WCA achieves highly accurate optimal solutions within merely 117 to 300 iterations, whereas PSO and GA require nearly 1000 iterations or get trapped in local optima. In practical electromagnetic design, this rapid convergence characteristic drastically reduces the number of time-consuming full-wave simulations, thereby substantially saving overall computational time and resources.

### 2.3. WCA-Based Antenna Optimization Scheme

**Figure 4:**
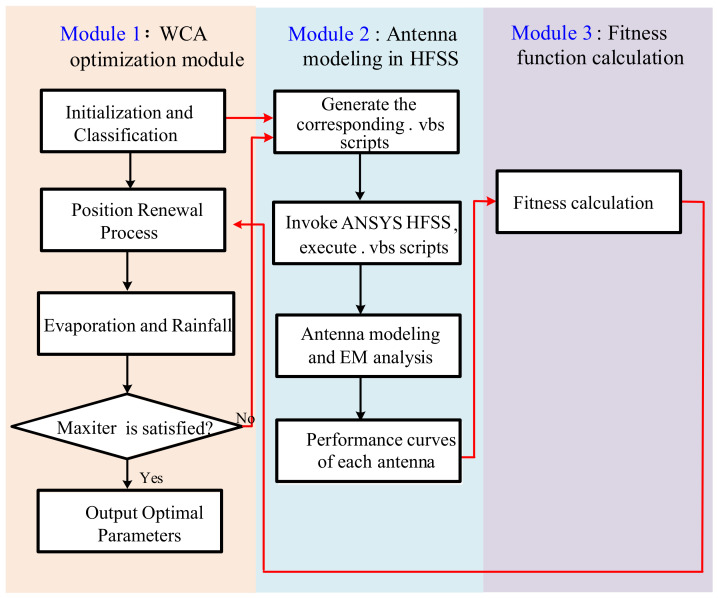
Flowchart of the WCA-based antenna optimization scheme.

Figure 4 provides the specific flowchart of the WCA-based antenna optimization scheme, which includes three modules. Module 1: WCA optimization module; Module 2: antenna modeling in HFSS; and Module 3: fitness function calculation. Herein, it should be highlighted that the ANSYS HFSS workbench is combined with the MATLAB software via HFSS-MATLAB-API. In the following, these three modules are illustrated in detail.

#### 2.3.1. Module 1: WCA Optimization Module

Firstly, enough raindrops are initialized to cover the problem space. Each raindrop contains the parameters of the desired antenna structure, and each parameter is located in a proper interval. Obviously, the dimension of each raindrop is determined by the number of parameters. The information contained in each raindrop will be imported into the ANSYS HFSS software to form the corresponding antenna structure in Module 2. Based on the fitness value calculated in Module 3, the total raindrops are divided into the sea, rivers and streams. Subsequently, the position update implementation is performed to lead the streams to flow to the sea and rivers, and rivers to flow to the sea. The evaporation and rainfall procedures will be carried out once the designated constraints are satisfied. When the maximum number of iterations *N*_iter_ is reached, the optimization is terminated.

#### 2.3.2. Module 2: Antenna Modeling in HFSS

With the help of HFSS-MATLAB-API, the codes written with MATLAB software in Module 1 can be converted into a series of .vbs scripts, each of which can be executed in ANSYS HFSS to establish a corresponding antenna structure. After the EM analysis in ANSYS HFSS, the performance curves of each antenna can be obtained, such as the S-parameter and the antenna pattern at some given frequencies, which will be imported into Model 3 to assess the performance of each antenna.

#### 2.3.3. Module 3: Calculation of Fitness Value

A proper fitness function should be designed to assess the performance of each antenna simulated in Module 2. Based on the assessment in this module, the sea, rivers and streams are updated in Module 1. In the following two sections, the applications of WCA in antenna design and array antenna optimization are provided, respectively.

## 3. WCA-Based Antenna Design

In this section, the performance of WCA in antenna design is firstly evaluated by designing two typical antennas, i.e., a dual-band E-shaped patch antenna and a conventional ME dipole antenna, and is further examined by designing a bandwidth-enhanced ME dipole antenna with a simple structure. The corresponding simulated parameters of WCA are set as β = 2, $K_{r}$ = 3, τ = 1 × 10^−16^, and σ = 0.1. Different bandwidth evaluation criteria are adopted for each antenna to ensure direct comparability with the benchmark results in the cited literature.

### 3.1. Design of E-Shaped Patch Antenna

**Figure 5:**
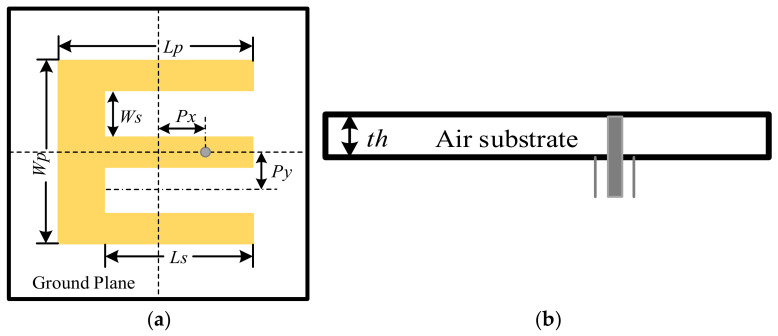
Geometry of E-shaped patch antenna. (**a**) Top view and (**b**) side view.

The E-shaped patch antenna is a typical example of patch antennas exhibiting enlarged operational bandwidth. To evaluate the performance of the WCA in antenna design, an optimization design on a dual-band E-shaped patch antenna is first implemented. Figure 5 provides the geometry of an E-shaped patch antenna, where the parameters are also defined. As depicted in Figure 5, the E-shaped patch antenna consists of an E-shaped patch, which is formed by symmetrically etching two identical slots out of a rectangular patch with dimensions of *Lp* × *Wp*, a ground with a size of 60 mm × 60 mm, and a 50 Ω coaxial probe, where the E-shaped patch is located 5 mm above the ground. The dual-frequency characteristic of the E-shaped antenna originates from two parallel slots on the patch. These symmetric slots fundamentally alter the surface current distribution and excite two independent resonant modes by establishing distinct current paths along the central and outer arms, thereby effectively broadening the operating bandwidth. Since the slot dimensions and feed position strictly determine the resonant frequencies, WCA is utilized to optimize these key geometric parameters. This precise tuning ensures multiple well-matched resonances for optimal dual-band performance.

The E-shaped patch antenna is expected to operate at the two frequencies of 5.0 GHz and 5.5 GHz. Therefore, the goal is to minimize the |*S*_11_| parameters at these two frequencies. As depicted in Figure 5, six parameters, i.e., *Wp*, *Lp*, *Ws*, *Ls*, *Px*, and *Py*, are included in the optimization since these parameters can significantly affect the performance of the antenna. The searching space, i.e., the minimum and maximum values of the six parameters, is listed in Table 3 to maintain the E-shaped structure during the optimization procedure, which is the same as those in [4,28].

According to the above descriptions, the fitness function is established as

(11) $$\underset{R}{\mathrm{minimize}}\;f(R)=\max\left\{S_{11}^{5.0}\;,\;S_{11}^{5.5}\right\}$$

where $R\;=\;{\left[Wp,\;Lp,\;Ws,\;Ls,\;Px,\;Py\right]}^{T}$ is the raindrop vector, the operator max(⋅) represents the maximum value extraction. It should be emphasized that the max operator here is only used to select the larger S_11_ value at the two operating frequencies of 5.0 GHz and 5.5 GHz, representing the worst impedance matching performance. The optimization goal is to minimize this maximum value, so that both frequencies can achieve good impedance matching simultaneously. In each optimization process, the maximum number of iterations $N_{iter}$ is set as 50. In the WCA, the raindrop population size *K* balances global exploration and local exploitation. An excessively large *K* imposes an unacceptable computational burden due to time-consuming full-wave electromagnetic simulations, while a small *K* insufficiently samples the highly nonlinear problem space, leading to premature convergence to local optima. To effectively balance optimization accuracy and efficiency, *K* is set to 20 in this study.

**Figure 6:**
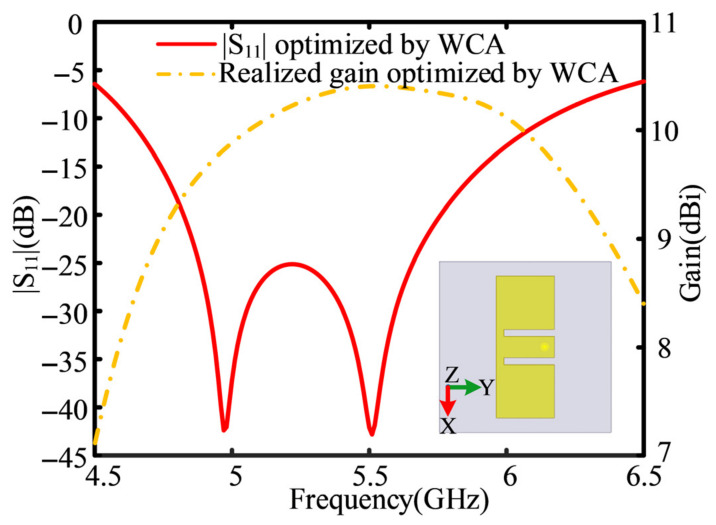
Simulated |*S*_11_| and realized gain curves of the optimized E-shaped patch antenna.

The optimization is executed 20 times independently. The optimal result with the widest operational bandwidth is selected, and the related antenna parameters are provided in Table 4. The associated *S*_11_ curve and the realized gain curve of the resultant E-shaped patch antenna are presented in Figure 6, along with the final antenna structure. As can be seen from Figure 6, the values of S_11_ are −37.18 dB at 5.0 GHz and −42.11 dB at 5.5 GHz, which are better than those obtained with GWO [4], DE [20], SaDE, and WDO [28]. The WCA optimization focuses on six key geometric parameters, precisely adjusting the resonant length and feed coupling strength. Such precise parameter tuning achieves strong impedance matching at the two target frequencies. Note that the searching space is identical for all of these methods during the optimization. Detailed maximum values of *S*_11_ at the two frequencies are provided in Table 5, along with the obtained operational bandwidths with these methods. It can be seen from Table 5 that the maximum 10 dB bandwidth of 28.41% is achieved with WCA ranging from 4.62 GHz to 6.15 GHz.

To further evaluate the performance of the proposed WCA-based scheme, statistical results of 20 independent runs are analyzed in Table 6. The best, worst, mean and standard deviation of the optimized fitness values are summarized in Table 6. The small variation between the best and worst fitness values, alongside a low standard deviation, demonstrates the remarkable stability and robustness of the WCA in electromagnetic optimization.

### 3.2. Design of Conventional ME Dipole Antenna

**Figure 7:**
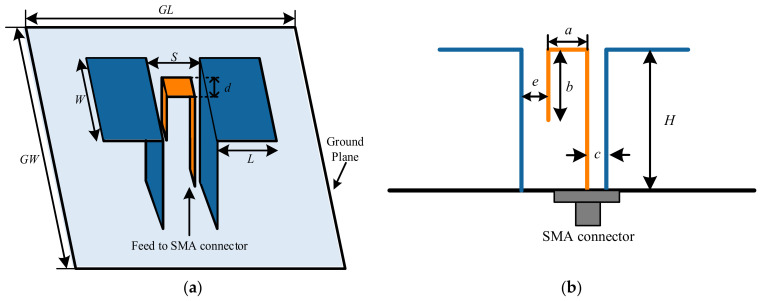
Geometry of conventional ME dipole antenna [38]; (**a**) 3D view and (**b**) side view.

The conventional ME dipole antenna [38] is selected as the second typical antenna to further verify the effectiveness of WCA in the design of antennas since it exhibits a quite wide bandwidth and a stable radiation pattern simultaneously. Figure 7 depicts the geometry of a conventional ME dipole antenna, along with the parameter description. The wideband characteristic of this antenna is achieved by the combined resonance of its electric and magnetic dipole structures. As is shown in Figure 7, the ME dipole antenna consists of a planar dipole and a vertically oriented quarter-wave shorted patch. A Γ-shaped probe is adopted to excite these two orthogonally placed structures simultaneously, and a ground plane is utilized to reduce the back radiation. The width and length of each planar patch are *W* and *L*, respectively. The shorted patch antenna has a height of *H*. The two vertical patches are separated by a gap of *S*. The Γ-shaped probe is composed of three portions, i.e., the right vertical patch, the horizontal patch and the left transmission line. The four parameters, *a*, *b*, *c*, and *d*, are adopted to describe the Γ-shaped probe. By optimizing the key structural parameters using the WCA, the two resonant points are further broadened and merged smoothly, which significantly extends the operating bandwidth while maintaining stable gain and low cross-polarization levels.

In the optimization procedure, the dimensions of the ground plane are fixed as *GW* × *GL* = 120 mm × 120 mm. From Figure 7, it is obviously observed that some constraints need to be satisfied to ensure that the ME dipole can work successfully, i.e., *b* < *H*, *a* < *S*, *c* < *S* and *a* + *c* < *S*. However, these constraints are difficult to realize since the values of *a*, *c*, *S*, and *H* are randomly generated in the iterative optimization procedure. Moreover, if the above constraints are added to the program, running time and computational complexity would be increased greatly. A new parameter *e* is introduced in [4] to cleverly simplify the optimization problem, which indicates the distance between the left vertical patch and the Γ-shaped strip feed line. The same way is also utilized in our work. As can be seen from Figure 7b, the relationship among the parameters *a*, *e*, *c*, and *S* satisfies the following equation.

(12) S=e+a+c

The ME dipole is supposed to cover the frequency region from 1.5 GHz to 3.5 GHz, with the center frequency located at 2.5 GHz. Based on the above analysis, the search space of the ME dipole antenna during the optimization process is listed in Table 7.

The optimization goal is to broaden the bandwidth; thus, the fitness function is set as

(13) $$\underset{R}{\mathrm{minimize}}\;\underset{f\in [f_{L},f_{H}]}{f(R)}=\max\left({\mathrm{VSWR}}_{\;\mathrm{from}\;1.5\mathrm{GHz}\;\mathrm{to}\;3.5\mathrm{GHz}}\right)$$

where $R={\left[W,\;L,\;H,\;a,\;b,\;c,\;d,\;e\right]}^{T}$ is the design variable vector, and the optimization objective is to minimize the maximum voltage standing wave ratio(VSWR) over the operating band. With the same parameter settings as in Section 3.1, a fitness value of 1.55 is achieved after 20 iterations. The ME dipole antenna with the widest bandwidth is selected and its corresponding parameter values are provided in Table 8.

**Figure 8:**
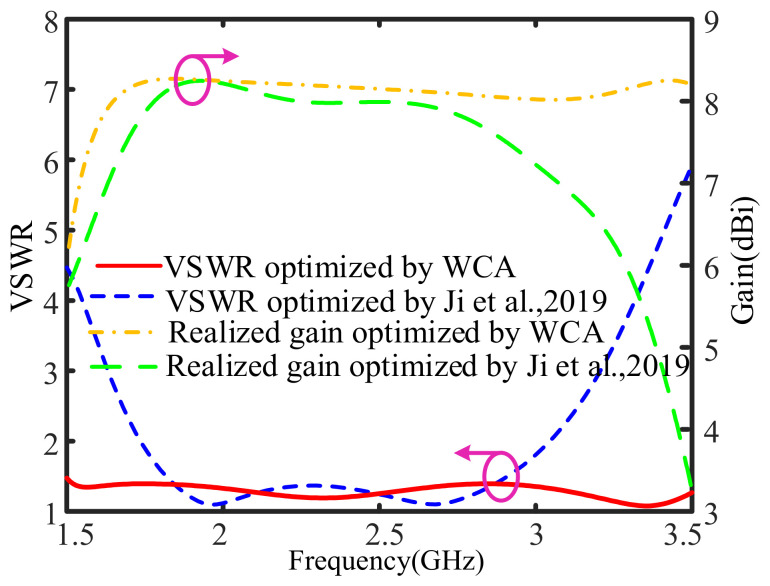
Simulated VSWR and realized gain curve of the optimized ME dipole antenna [35].

**Figure 9:**
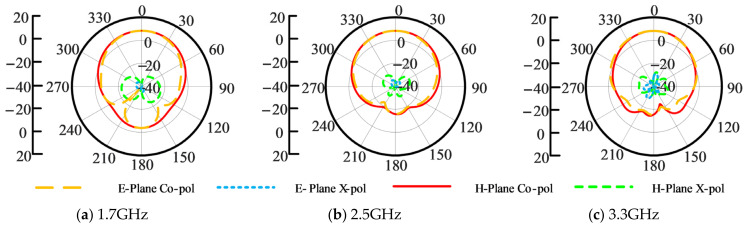
Simulated radiation patterns in E- and H-planes of the optimized ME dipole antenna at different frequencies; (**a**) 1.7 GHz, (**b**) 2.5 GHz and (**c**) 3.3 GHz.

The VSWR and gain curves of the optimized ME dipole via WCA and those of the ME dipole presented in [38] are provided in Figure 8. The optimized ME dipole exhibits a wide bandwidth of 80.9% for VSWR ≤ 1.5 (from 1.51 GHz to 3.56 GHz) and 87.2% for VSWR ≤ 2 (from 1.45 GHz to 3.69 GHz). An average gain of 8.1 dBi is maintained across the operational region. Figure 9 provides the simulated radiation patterns in E- and H-planes of the optimized ME dipole antenna at 1.7 GHz, 2.5 GHz and 3.3 GHz. From this figure, it is seen that the cross-polarization radiation levels at 1.7 GHz, 2.5 GHz, and 3.3 GHz are all less than −23 dB. The patterns in both E- and H-planes are generally symmetrical and exhibit good unidirectional radiation characteristics.

Statistical results obtained from 20 independent optimization runs are presented in Table 9 to characterize the optimization stability of the WCA for the conventional ME dipole antenna. The best, worst, mean, and standard deviation of the maximum VSWR and the relative bandwidth (VSWR ≤ 1.5) are summarized. The slight fluctuations in the statistical results validate the outstanding stability and robustness of the WCA for wideband antenna optimization.

The detailed results, including bandwidth and antenna size, are summarized in Table 10. As shown in Figure 8 and Table 10, compared with the optimal results in [38], the operational bandwidth of the ME dipole antenna optimized by WCA is widened by 84.7%. It is worth mentioning that the antenna size is effectively reduced by 25.9%. Compared with the optimal results in [4], the bandwidth of the ME dipole optimized by WCA is slightly improved, while the antenna size is decreased by 31.6%. To sum up, competitive optimal results can be achieved with WCA in terms of bandwidth, gain, radiation pattern and size.

### 3.3. Design of Bandwidth-Enhanced ME Dipole Antenna

**Figure 10:**
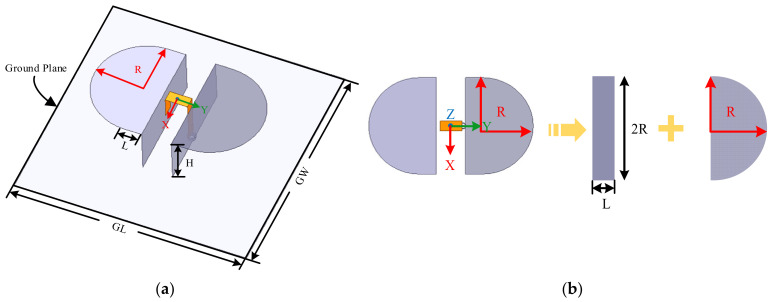
Geometry of proposed ME dipole antenna; (**a**) 3D view and (**b**) top view.

To further verify the effectiveness of WCA in antenna design, a bandwidth-enhanced ME dipole featuring a simple structure is proposed in Figure 10. The electric dipole combines a rectangular and a semicircular patch. This smooth contour of the semicircular patch alleviates impedance discontinuities, introduces multiple adjacent resonant modes, and extends the effective surface current path, thereby significantly broadening the overall impedance bandwidth. As an illustration, the top view of the proposed antenna is presented in Figure 10b. The dimensions of the rectangular patch are expressed as (*L*, 2*R*) where *R* denotes the radius of the semicircular patch. Other antenna parameters including *GW*, *GL*, *H*, *a*, *b*, *c*, *d*, and *e* are also utilized to denote the common parts of ME dipole antenna depicted in Section 3.2.

The searching dimensions of the ME dipole antenna with enhanced bandwidth are provided in Table 11. The center frequency of the ME dipole antenna is set to 2.6 GHz.

Similar to Section 3.2, to achieve a wide bandwidth, the fitness function is defined as

(14) $$\underset{R}{\mathrm{minimize}}\;\underset{f\in [f_{L},f_{H}]}{f(R)}=\max\left({\mathrm{VSWR}}_{\;\mathrm{from}\;1.4\mathrm{GHz}\;\mathrm{to}\;3.8\mathrm{GHz}}\right)$$

Similarly, the optimization goal here is to minimize this fitness function. By minimizing the maximum VSWR value across the entire target frequency band, the WCA effectively suppresses the peak reflection coefficient, thereby achieving stable impedance matching and enhanced bandwidth. In the optimization procedure, the size of the population and the maximum number of iterations are also set as 20 and 50, respectively, referring to the two design examples above. After 20 independent iterations, the best result with the widest bandwidth is chosen, and the corresponding dimensions of the resultant antenna are provided in Table 12.

**Figure 11:**
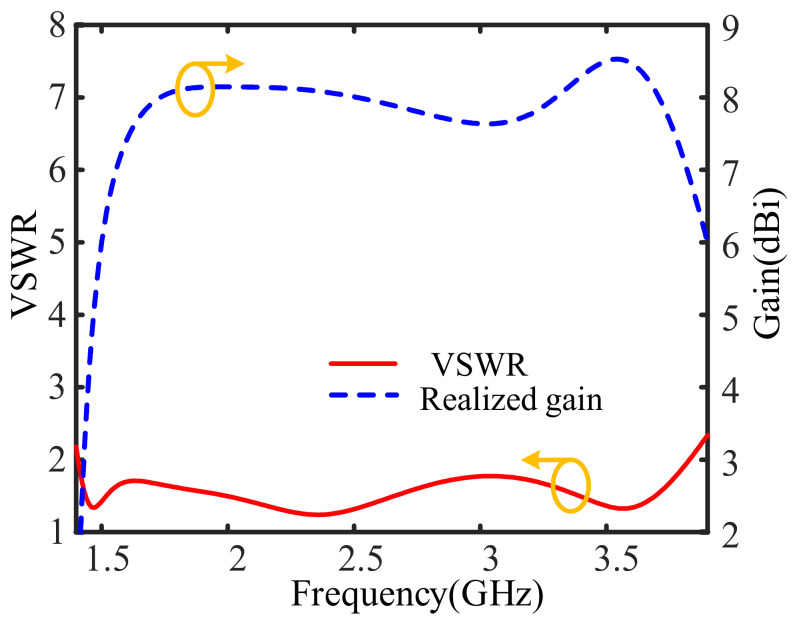
Simulated VSWR and realized gain curves of the bandwidth-enhanced ME dipole antenna.

**Figure 12:**
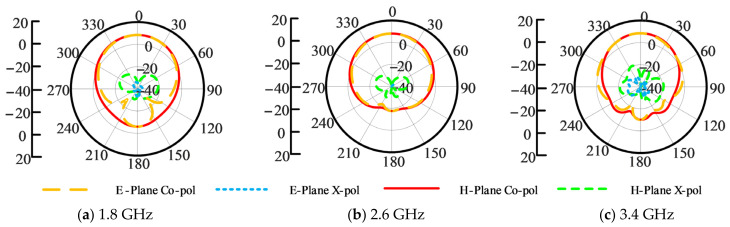
Simulated radiation patterns of the optimized ME dipole antenna with enhanced bandwidth at different frequencies.

The simulated VSWR and gain curves are provided in Figure 11. It can be seen that a bandwidth of 92.4% (ranging from 1.41 GHz to 3.83 GHz) in terms of VSWR ≤ 2 is realized. Also, an antenna gain of around 8.0 dBi is achieved. Figure 12 provides the simulated radiation patterns at frequencies of 1.8 GHz, 2.6 GHz, and 3.4 GHz, respectively. From Figure 12, it is seen that the cross-polarization levels at these frequencies are all lower than −25 dB. Almost equal radiation patterns in E- and H-planes are obtained.

Table 13 provides the performance comparisons of the proposed ME dipole antenna with other reported ME dipole antennas. At present, many methods have been reported to enhance the bandwidth of the ME dipole antenna. Herein, four typical attempts are provided for comparison, including utilizing an E-shaped patch in lieu of the rectangular patch [39], adopting dual open-ended slot excitation [40], microstrip line aperture-coupled excitation [41], and defected ground structure [42]. As can be seen from Table 13, the designed ME dipole with the help of WCA achieves a wider bandwidth. This design example further demonstrates that WCA is a competitive candidate in antenna design.

Similarly, the statistical performance of 20 independent runs for the bandwidth-enhanced ME dipole antenna is analyzed in Table 14 to evaluate the convergence reliability of the algorithm. The stable convergence behavior observed in these repeated tests indicates that the WCA maintains strong robustness while achieving wider impedance bandwidth performance.

## 4. WCA-Based Low-SLL Beampattern Synthesis

In this section, the WCA is applied for the low-SLL beampattern synthesis based on a uniformly spaced (*d* = 50 mm, 0.5*λ* at 3.0 GHz) 27-element array antenna, where a folded fractal ME dipole is designed as the element. The descriptions of the 27-element folded fractal ME dipole array antenna are provided in Section 4.1, and the WCA-based low-SLL beampattern synthesis is presented in Section 4.2.

### 4.1. Design of Folded Fractal ME Dipole Array Antenna

**Figure 13:**
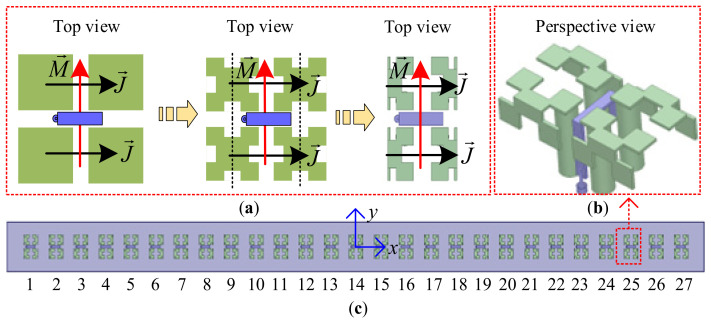
Configuration of a uniformly spaced 27-element folded fractal ME dipole array antenna. (**a**) Design procedures of fractal ME dipole antenna, (**b**) perspective view of designed fractal ME dipole antenna and (**c**) uniformly spaced 27-element array antenna.

Figure 13 provides the configuration of the uniformly spaced 27-element folded fractal ME dipole array antenna, where the design procedures are provided in Figure 13a. To reduce the aperture size of the four-sectional ME dipole antenna, the 1st order Minkowski structure is firstly adopted for each horizontal patch, and then each fractal patch is folded along its symmetric line. Figure 13b presents the perspective view of the designed folded fractal ME dipole antenna, and its aperture size is 26.4 mm × 47.8 mm (0.264*λ* × 0.478*λ* at 3.0 GHz). Then this antenna is uniformly arranged to form a 27-element array antenna with element spacing of 50 mm (0.5*λ* at 3.0 GHz), as depicted in Figure 13c.

**Figure 14:**
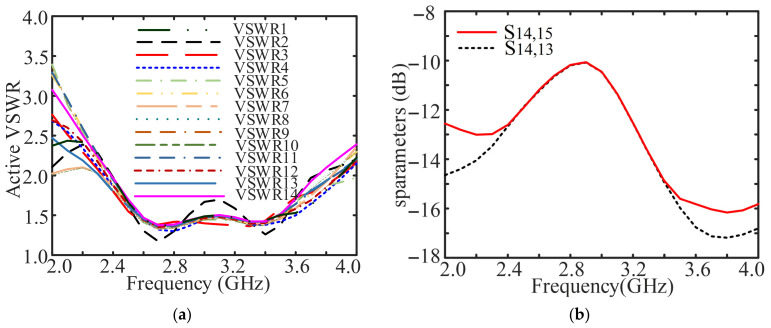
Simulated parameters of 27-element folded fractal ME dipole array antenna. (**a**) Active VSWRs and (**b**) S parameters.

Figure 14 provides the simulated active VSWRs from the 1st to the 14th elements, and s-parameters between the 14th element and its adjacent two elements. From Figure 14a, it can be seen that the overlapped bandwidth of 43.5% (from 2.41 GHz to 3.75 GHz) is achieved in terms of active VSWR < 2.0. Actually, the criterion of active VSWR < 2.5 is usually utilized to calculate the operational bandwidth of an array in engineering scenarios. Meanwhile, it is seen from Figure 14b that the maximum mutual coupling can be as high as −10 dB.

### 4.2. Low-SLL Beampattern Synthesis Based on Folded Fractal ME Dipole Array Antenna

According to array antenna theory, the electric pattern of the 27-element folded fractal ME dipole array antenna in the far-field region can be formulated as

(15) $$F\left(\theta ,\varphi \right)=\sum_{n=1}^{N}EP_{n}(\theta ,\varphi )I_{n}e^{j{\vec{k}}^{T}{\vec{r}}_{n}}$$

where $EP_{n}(\theta ,\varphi )$, $I_{n}$ and ${\vec{r}}_{n}={\left[\left(n-1\right)d,0,0\right]}^{T}$, *n* = 1, …, *N* (*N* = 27 herein), respectively denote the element pattern, exciting current and position vector of the *n*-th element, and $\vec{k}=\frac{2\pi }{\lambda }{\left[\sin\left(\theta \right)\cos\left(\varphi \right),\sin\left(\theta \right)\sin\left(\varphi \right),cos\left(\theta \right)\right]}^{T}$ is the wave vector. Note that $EP_{n}(\theta ,\varphi )$ is different from its counterpart, i.e., EP(θ,φ), in an isolated situation. Express $EP_{n}(\theta ,\varphi )$ in the following form

(16) $$EP_{n}(\theta ,\varphi )=\xi_{n}\left(\theta ,\varphi \right)EP(\theta ,\varphi )$$

where $\xi_{n}\left(\theta ,\varphi \right)$ denotes the complex-valued coefficient induced by the mutual coupling effects.

Therefore, Equation (15) can be further written as

(17) $$\begin{array}{ll} F\left(\theta ,\varphi \right) & =EP(\theta ,\varphi )\sum_{n=1}^{27}\xi_{n}(\theta ,\varphi )I_{n}e^{j{\vec{k}}^{T}{\vec{r}}_{n}}\\ & =EP(\theta ,\varphi )I^{H}s_{a}\left(\theta ,\varphi \right) \end{array}$$

where $I={\left[I_{1},\cdots ,I_{n},\cdots ,I_{N}\right]}^{H}$ is the exciting current vector with the superscript being the conjugate transpose operator, and $s_{a}\left(\theta ,\varphi \right)=m⊙s\left(\theta ,\varphi \right)$ is the actual steering vector of the array with ⊙ being the Schur–Hadamard product; $m={\left[\xi_{1},\cdots ,\xi_{n},\cdots ,\xi_{N}\right]}^{T}$ denotes the mutual coupling coefficient vector and $s\left(\theta ,\varphi \right)={\left[e^{j{\vec{k}}^{T}{\vec{r}}_{1}},\cdots ,e^{j{\vec{k}}^{T}{\vec{r}}_{n}},\cdots ,e^{j{\vec{k}}^{T}{\vec{r}}_{N}}\right]}^{T}$ is the ideal steering vector.

To verify the effectiveness of WCA in array synthesis, the low-SLL synthesis is chosen as an illustration. Specifically, the fitness function is constructed based on the criterion of minimizing the maximum SLL, i.e.,

(18) $$\underset{I}{\mathrm{minimize}}\;f(I)=\max\left(I^{H}S_{a}I\right)$$

where $S_{a}=s_{a}\left(\theta ,\varphi \right){s_{a}}^{H}\left(\theta ,\varphi \right)$ denotes the actual covariance matrix of the array in the sidelobe region. It should be emphasized that the max(⋅) operator here is used to select the PSLL within the designated sidelobe region. The optimization goal is to minimize this maximum value via the WCA, thereby effectively suppressing the worst-case sidelobe level and achieving the desired low-SLL beampattern.

In practical array environments, antenna elements interact with each other through electromagnetic fields, resulting in non-negligible mutual coupling. It should be emphasized that mutual coupling only refines the electromagnetic forward model and does not change the intrinsic optimization mechanism or performance of the WCA. Neglecting mutual coupling will introduce obvious deviations between the ideal fitness evaluation and the actual electromagnetic response. By embedding the mutual coupling effects into the fitness function via the actual covariance matrix, a more accurate and practical electromagnetic forward model is established, which effectively reduces the model error and makes the fitness evaluation more consistent with real engineering scenarios.

**Figure 15:**
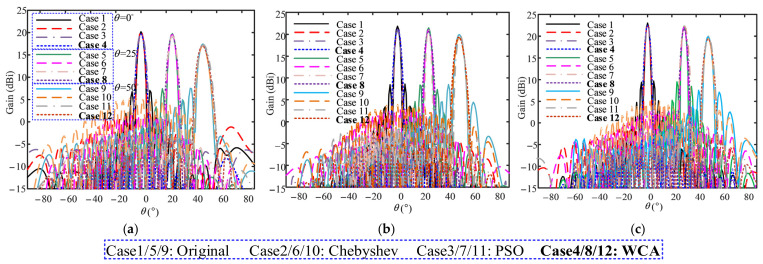
Simulated beampatterns at (**a**) 2.5 GHz, (**b**) 3.0 GHz, and (**c**) 3.5 GHz under $\theta =0^{\circ }$, $\theta \;{=25}^{\circ }$ and $\theta =50^{\circ }$ beam steering, where Cases 1–4 correspond to $\theta =0^{\circ }$; Cases 5–8 to $\theta \;{=25}^{\circ }$; Cases 9–12 to $\theta =50^{\circ }$; each group includes original, −20 dB Chebyshev-weighted, PSO- and WCA-synthesized patterns.

Figure 15 provides the original and synthesized beampatterns at 2.5 GHz, 3.0 GHz, and 3.5 GHz of the 27-element folded fractal ME dipole array antenna, where the beam is steered to 0°, 25°, and 50° as illustrated. Note that the beampatterns obtained with the conventional PSO algorithm and the −20 dB Chebywin tapering weight are also provided as comparisons in each beam steering as well as the original beampattern. Table 15, Table 16 and Table 17 provide the corresponding gains and SLLs at these three frequencies in different cases. As summarized in Table 15, Table 16 and Table 17, the −20 dB Chebyshev weighting achieves an average SLL reduction of 4.2 dB, while PSO achieves an average reduction of 10.8 dB. In comparison, the proposed WCA-based method achieves an average SLL reduction of 13.7 dB across all tested frequencies and beam steering angles, outperforming PSO by 2.9 dB on average. The maximum SLL reduction reaches 16.4 dB at 3.5 GHz with the beam steered to 0°. Notably, all three methods maintain excellent gain retention while suppressing sidelobes, with only marginal gain degradation. In summary, WCA exhibits superior performance in low-SLL synthesis in the presence of strong mutual coupling effects, which verifies the applicability and high efficiency of the algorithm to practical array pattern synthesis problems with coupling effects.

To further evaluate the computational efficiency and convergence speed of the proposed method, Table 18 provides a detailed comparison between the standard PSO and the WCA at a beam steering angle of 0° across three different frequencies. The results clearly demonstrate that the proposed WCA consistently outperforms the PSO algorithm across all tested frequencies. Specifically, WCA achieves faster convergence speed with significantly reduced computational time. For instance, at the center frequency of 3.0 GHz, WCA completes the optimization in only 0.65 s, whereas PSO requires 1.82 s. Meanwhile, WCA converges within 253 iterations, which is substantially fewer than the 497 iterations needed by PSO. It should be noted that the Chebyshev weighting method is an analytical closed-form approach with no iterative optimization process, and is therefore not included in this computational efficiency comparison for iterative optimization algorithms. These findings validate that the proposed WCA possesses lower computational complexity and higher efficiency.

## 5. Conclusions

An application innovation is presented by introducing the WCA for antenna design and array antenna synthesis in this paper. Specifically, two representative antennas, i.e., a dual-band E-shaped patch antenna and a typical ME dipole antenna, are firstly designed to demonstrate its effectiveness in antenna design. Comparisons with some well-known typical metaheuristic methods, such as GA, PSO and DE are provided, from which it is seen that better or competitive results can be achieved. Then a bandwidth-enhanced ME dipole antenna is designed with simple structure based on WCA. In the sequel, the WCA-based low-SLL synthesis is implemented based on a 27-element folded fractal ME dipole array antenna, which further demonstrates the effectiveness of WCA in array synthesis.

## Figures and Tables

**Table 1:** Benchmark functions.

Function Names	Expressions	Dimension(D)	Boundaries	Optimal Solution
Sphere function(F_1_)	$f_{1}\left(x\right)=\sum_{i=1}^{D}{x_{i}}^{2}$	10, 20, 30	$-100\leq x_{i}\leq 100$	$x^{*}=\left[0,0,\ldots ,0\right],\;f_{1}\left(x^{*}\right)=0$
Schwefel’s Problem 2.22 (F_2_)	$f_{2}\left(x\right)=\sum_{i=1}^{D}\left|x_{i}\right|+\prod_{i=1}^{D}\left|x_{i}\right|$	10, 20, 30	$-10\leq x_{i}\leq 10$	$x^{*}=\left[0,0,\ldots ,0\right],\;f_{2}\left(x^{*}\right)=0$
Ackley’s Function (F_3_)	$\begin{array}{ll} f_{3}\left(x\right)= & -20\exp\left(-0.2\sqrt{\frac{1}{D}\sum_{i=1}^{D}{x_{i}}^{2}}\right)-\exp\left(\frac{1}{D}\sum_{i=1}^{D}\cos2\pi x_{i}\right)\\ & +20+e \end{array}$	10, 20, 30	$-32\leq x_{i}\leq 32$	$x^{*}=\left[0,0,\ldots ,0\right],\;f_{3}\left(x^{*}\right)=0$
Generalized Penalized Function (F_4_)	$\begin{array}{l} f_{4}\left(x\right)=\frac{\pi }{D}\{10\sin^{2}\left(\pi y_{1}\right)+\sum_{i=1}^{D-1}{\left(y_{i}-1\right)}^{2}\\ \;\cdot \left[1+10\sin^{2}\left(\pi y_{i}+1\right)\right]+{\left(y_{D}-1\right)}^{2}\}\;+\sum_{i=1}^{D}u\left(x_{i},10,100,4\right) \end{array}$ where $u\left(x_{i},a,k,m\right)=\left\{\begin{array}{ll} k{\left(x_{i}-a\right)}^{m}, & x_{i}>a,\\ 0, & -a\leq x_{i}\leq a\\ k{\left(-x_{i}-a\right)}^{m}, & x_{i}<-a. \end{array}\right.,$ and $y_{i}=1+\frac{1}{4}\left(x_{i}+1\right).$	10, 20, 30	$-50\leq x_{i}\leq 50$	$x^{*}=\left[1,1,\ldots ,1\right],\;f_{4}\left(x^{*}\right)=0$

**Table 2:** Comparisons of PSO, GA and WCA.

Function	Dimension	Algorithm	Best	Worst	Mean	Std	Time(s)	Convergence Iter
F_1_	30	PSO	1.4 × 10^−1^	3.9 × 10^−1^	2.1 × 10^−1^	5.0 × 10^−2^	2.7375	989
		GA	3.1 × 10^−3^	6.6 × 10^−2^	2.2 × 10^−2^	1.5 × 10^−2^	2.9874	938
		WCA	**3.7 × 10^−20^**	**2.0 × 10^−14^**	**1.3 × 10^−15^**	**3.8 × 10^−15^**	**1.0754**	**122**
F_2_	30	PSO	9.2 × 10^−1^	1.6 × 10^0^	1.4 × 10^0^	1.7 × 10^−1^	2.7418	778
		GA	7.4 × 10^−1^	4.7 × 10^0^	1.9 × 10^0^	8.6 × 10^−1^	2.9937	971
		WCA	**5.4 × 10^−11^**	**2.3 × 10^−8^**	**4.4 × 10^−9^**	**5.7 × 10^−9^**	**1.1503**	**167**
F_3_	30	PSO	1.3 × 10^0^	1.8 × 10^0^	1.6 × 10^0^	1.4 × 10^−1^	3.2154	659
		GA	2.2 × 10^0^	5.5 × 10^0^	3.6 × 10^0^	8.5 × 10^−1^	3.2158	993
		WCA	**1.8 × 10^−13^**	**5.0 × 10^−4^**	**1.3 × 10^−5^**	**7.9 × 10^−5^**	**1.5203**	**305**
F_4_	30	PSO	1.8 × 10^0^	1.9 × 10^0^	1.9 × 10^0^	1.6 × 10^−2^	3.8280	893
		GA	9.5 × 10^−7^	8.3 × 10^−1^	1.1 × 10^−1^	2.1 × 10^−1^	3.8832	881
		WCA	**5.8 × 10^−21^**	**1.0 × 10^−2^**	**2.6 × 10^−3^**	**2.6 × 10^−2^**	**2.0306**	**186**

**Table 3:** Searching space of E-shaped patch antenna.

**Parameters**	***Wp***	***Lp***	***Ws***
Min (mm)	10	10	0.5
Max (mm)	50	30	*Wp*/2
**Parameters**	***Ls***	***P_x_***	***Py***
Min (mm)	0.5	−*Lp*/2	*Ws*/2
Max (mm)	*Lp*	*Lp*/2	*Wp*/2−*Ws*/2

**Table 4:** Optimized parameters of E-shaped patch antenna.

Parameters	*Wp*	*Lp*	*Ws*	*Ls*	*Px*	*Py*
Values(mm)	49.79	20.29	2.47	17.49	4.96	6.77

**Table 5:** Results comparison for the design of the E-shaped patch antenna with different methods.

Ref.	Methods	Max.S_11_ (dB)	BW (%)
[4]	GWO	−34.59	26.73
[20]	DE	−30.48	20.55
[20]	SaDE	−34.06	20.18
[28]	WDO	−31.00	20.00
Prop.	**WCA**	**−37.18**	**28.41**

**Table 6:** Statistical performance of the WCA for the E-shaped patch antenna.

Metric	Best	Worst	Mean	Std
Max.S_11_ (dB)	−37.18	−26.26	−34.72	2.24
Bandwidth (%)	28.41	26.35	27.68	0.48

**Table 7:** Searching space of the ME dipole antenna.

Parameters	*W*	*L*	*H*	*a*	*b*	*c*	*d*	*e*
Min (mm)	30	10	10	4.5	12	0.5	2	1.5
Max (mm)	90	50	50	14.5	32	5	8	11.5

**Table 8:** Optimized parameters of the conventional ME dipole antenna.

**Parameters**	***W***	***L***	***H***	***a***	***b***
Values (mm)	53.57	27.77	27.20	10.53	14.34
**Parameters**	***c***	***d***	***e***	***S***	
Values (mm)	1.89	5.68	1.93	14.35	

**Table 9:** Statistical performance of the WCA for the conventional ME dipole antenna.

Metric	Best	Worst	Mean	Std
Max. VSWR	1.45	1.49	1.47	0.02
Bandwidth (%)	80.9	78.5	79.5	0.76

**Table 10:** Results comparison for the design of ME dipole antenna with different methods.

Ref.	Method	Parameters (mm)				Antenna Size	BW (%) (VSWR ≤ 1.5)
		*W*	*L*	*H*	*S*		
[38]	HFSS	60	30	30.0	17	0.50*λ* × 0.64*λ* × 0.250*λ*	43.8
[4]	GWO	74.23	28.01	27.91	15.76	0.62*λ* × 0.60*λ* × 0.233*λ*	80.0
**Prop.**	WCA	53.57	27.77	27.20	14.35	**0.45*λ* × 0.58*λ* × 0.227*λ***	**80.9**

where *λ* denotes the wavelength corresponding to the center operating frequency, i.e., 2.5 GHz.

**Table 11:** Searching space of the proposed ME dipole antenna.

Parameters	*R*	*L*	*H*	*a*	*b*	*c*	*d*	*e*
Min (mm)	15	10	10	4.5	12	0.5	2	1.5
Max (mm)	45	30	50	14.5	32	5	8	11.5

**Table 12:** Optimized dimensions of bandwidth-enhanced ME dipole.

**Parameters**	***R***	***L***	***H***	***a***	***b***
Values (mm)	24.31	20.67	26.36	12.26	12.04
**Parameters**	***c***	***d***	***e***	***S***	***/***
Values (mm)	1.93	5.11	2.2	16.39	***/***

**Table 13:** Performance comparison between the proposed and reported ME dipole antennas.

Ref.	Structure	Ave. Gain (dBi)	BW (%) (VSWR ≤ 2)
[39]	Complex	6.0	61.6
[40]	Simple	10.0	53.0
[41]	Simple	8.0	85.0
[42]	Simple	7.2	86.9
**Prop.**	**Simple**	**8.0**	**92.4**

**Table 14:** Statistical performance of the WCA for the bandwidth-enhanced ME dipole antenna.

Metric	Best	Worst	Mean	Std
Max. VSWR	1.65	1.90	1.76	0.08
Bandwidth (%)	92.4	89.1	90.7	0.90

**Table 15:** Gains and SLLs of beampatterns at 2.5 GHz in the above 12 cases.

Cases	Gain (dBi)	SLL (dB)	Cases	Gain (dBi)	SLL (dB)
Case 1	20.3	−13.6	Case 7	19.3	−23.2
Case 2	20.2	−18.4	**Case 8**	**19.2**	**−26.0**
Case 3	20.0	−24.3	Case 9	17.2	−9.8
**Case 4**	**19.8**	**−27.9**	Case 10	16.8	−12.4
Case 5	19.7	−12.3	Case 11	16.6	−18.6
Case 6	19.5	−16.2	**Case 12**	**16.4**	**−20.1**

**Table 16:** Gains and SLLs of beampatterns at 3.0 GHz in the above 12 cases.

Cases	Gain (dBi)	SLL (dB)	Cases	Gain (dBi)	SLL (dB)
Case 1	21.9	−14.3	Case 7	20.9	−25.6
Case 2	21.6	−19.7	**Case 8**	**20.7**	**−27.2**
Case 3	21.5	−24.1	Case 9	19.7	−11.5
**Case 4**	**21.3**	**−29.3**	Case 10	19.3	−15.4
Case 5	21.3	−13.1	Case 11	19.2	−21.3
Case 6	21.0	−18.2	**Case 12**	**19.0**	**−23.8**

**Table 17:** Gains and SLLs of beampatterns at 3.5 GHz in the above 12 cases.

Cases	Gain (dBi)	SLL (dB)	Cases	Gain (dBi)	SLL (dB)
Case 1	24.1	−13.6	Case 7	22.1	−25.8
Case 2	22.8	−19.2	**Case 8**	**21.9**	**−27.9**
Case 3	22.6	−27.4	Case 9	20.0	−12.6
**Case 4**	**22.5**	**−30.0**	Case 10	18.4	−14.5
Case 5	22.4	−12.9	Case 11	19.6	−20.2
Case 6	22.2	−17.9	**Case 12**	**19.3**	**−22.9**

**Table 18:** Computational time and convergence iteration comparison at θ = 0°.

Frequency (GHz)	Algorithm	Time(s)	Convergence Iterations
2.5	PSO	1.78	500
	**WCA**	**0.89**	**258**
3.0	PSO	1.82	497
	**WCA**	**0.65**	**253**
3.5	PSO	1.86	502
	**WCA**	1.08	**268**

## Data Availability

The original contributions presented in this study are included in the article. Further inquiries can be directed to the corresponding author.
